# A study combining microbubble-mediated focused ultrasound and radiation therapy in the healthy rat brain and a F98 glioma model

**DOI:** 10.1038/s41598-024-55442-6

**Published:** 2024-02-28

**Authors:** Stecia-Marie P. Fletcher, Amanda Chisholm, Michael Lavelle, Romy Guthier, Yongzhi Zhang, Chanikarn Power, Ross Berbeco, Nathan McDannold

**Affiliations:** 1 Department of Radiology, Brigham and Women’s Hospital, Boston, MA USA; 2 Department of Radiology, Harvard Medical School, Boston, MA USA; 3 Department of Radiation Oncology, Dana Farber Cancer Institute, Boston, MA USA; 4 Department of Radiation Oncology, Brigham and Women’s Hospital, Boston, MA USA; 5 Department of Radiation Oncology, Harvard Medical School, Boston, MA USA

**Keywords:** Preclinical research, Acoustics, Cancer therapy, CNS cancer, Biological physics

## Abstract

Focused Ultrasound (FUS) has been shown to sensitize tumors outside the brain to Radiotherapy (RT) through increased ceramide-mediated apoptosis. This study investigated the effects of FUS + RT in healthy rodent brains and F98 gliomas. Tumors, or striata in healthy rats, were targeted with microbubble-mediated, pulsed FUS (220 kHz, 102–444 kPa), followed by RT (4, 8, 15 Gy). FUS + RT (8, 15 Gy) resulted in ablative lesions, not observed with FUS or RT only, in healthy tissue. Lesions were visible using Magnetic Resonance Imaging (MRI) within 72 h and persisted until 21 days post-treatment, indicating potential applications in ablative neurosurgery. In F98 tumors, at 8 and 15 Gy, where RT only had significant effects, FUS + RT offered limited improvements. At 4 Gy, where RT had limited effects compared with untreated controls, FUS + RT reduced tumor volumes observed on MRI by 45–57%. However, survival benefits were minimal (controls: 27 days, RT: 27 days, FUS + RT: 28 days). Histological analyses of tumors 72 h after FUS + RT (4 Gy) showed 93% and 396% increases in apoptosis, and 320% and 336% increases in vessel-associated ceramide, compared to FUS and RT only. Preliminary evidence shows that FUS + RT may improve treatment of glioma, but additional studies are required to optimize effect size.

## Introduction

Glioblastoma Multiforme (GBM), a malignant grade IV glioma^1^, is an aggressive and lethal cancer of the brain and Central Nervous System (CNS). GBM is extremely common, accounting for 15.6% of all primary brain tumors and 45.2% of all primary malignant brain tumors in the United States^2^. The age-adjusted incidence rate of GBM is in the range of 2.5–4 per 100,000 persons^2^, and there is evidence that this number is increasing^3^. The current standard-of-care in the treatment of GBM is the Stupp Protocol, which involves maximal surgical resection, followed by radiation therapy (RT) with concomitant and adjuvant chemotherapy using temozolomide (TMZ)^4^. Even with the standard-of-care treatment, GBM frequently recurs, and prognosis remains poor, with a median survival of 15 months^5^ and a 5-year survival rate of < 5%^2^. There are a number of factors that contribute to poor prognosis in patients diagnosed with GBM, including the highly infiltrative nature of GBM, inter- and intra-tumor heterogeneity, the presence of the Blood–Brain Barrier (BBB), and the immunosuppressive environment in GBM^6^. Despite extensive efforts to develop new treatments, there are currently no curative treatment options for GBM patients^7,8^, and alternatives are desperately needed.

Focused ultrasound (FUS) is an emerging technology that allows the transcranial delivery of ultrasound waves to focal volumes in the brain, offering a non-invasive and precisely targeted therapeutic option. In the context of neuro-oncology, opportunities for FUS applications include BBB disruption to enhance drug delivery, thermal/mechanical ablation of brain tumors, radio-sensitization, and immunomodulation^9^. Czarnota et al. have demonstrated that microbubble- (MB-) mediated FUS can work synergistically with radiation therapy (RT) to decrease tumor growth and improve survival in mouse models of prostate, bladder, and breast cancer^10–14^. They have shown, through histologic analysis, that increased apoptosis is linked to increased levels of the sphingolipid, ceramide, on endothelial cells^15,16^. Ceramide plays an important role in the acid sphingomyelinase (ASMase) pathway for RT-induced apoptosis^17^. At high radiation doses (> ~ 15 Gy), RT acts directly to stress cell membranes, causing membrane alterations. This activates ASMase, which in turn generates ceramide, which acts as a secondary messenger for apoptosis. MB-mediated FUS + RT is currently in clinical trials for patients with head and neck cancer (NCT04431648) and breast cancer (NCT04431674). While this mechanism has not yet been investigated for CNS tumors, Chen et al., have recently demonstrated the synergistic effects of FUS + RT in a GL261 mouse model^18^ and there is an ongoing clinical trial investigating the safety and preliminary efficacy of FUS + RT using the NaviFUS system in patients with recurrent GBM (NCT04988750) . This group proposed that the mechanism for radiosensitization was increased perfusion and oxygenation post FUS. However, there is some evidence that FUS-mediated RT sensitization using oxygen MBs may lead to adverse effects in non-CNS tumors depending on the preclinical tumor model used^19^.

Beyond applications in GBM, FUS + RT may have applications in the management of other focal CNS diseases where RT is routinely used. These include brain metastases^20^ and non-malignant masses, like arteriovenous malformations (AVM)^21^. FUS may also be able to limit the RT dose in functional neurosurgery, where targeted, high-dose, radiosurgical procedures can be used to treat conditions such as Essential Tremor^22^ and Epilepsy^23^.

This study aimed to investigate the effects of FUS + MB-mediated RT (FUS + RT) in the brains of healthy rats and in rats bearing the F98 glioma model^24^. In the tumor model, the effects on tumor growth and survival, as well as histological markers of apoptosis and ceramide, were observed.

## Materials and methods

### Animals

All animal experiments were approved by the Institutional Animal Care and Use Committee (IACUC) at Brigham and Women’s Hospital. All methods were carried out in accordance with relevant guidelines and regulations. All study methods are reported in accordance with the Animal Research: Reporting of In Vivo Experiments (ARRIVE) guidelines.

Experiments in the healthy brain were performed in male Sprague-Dawley rats (~250 g, Charles River Laboratories, n = 19), while experiments in F98 tumor-bearing animals were performed in male Fischer rats (~ 250 g, Charles River Laboratories, n = 65). Animals were anesthetized with an intraperitoneal (IP) injection of ketamine (80 ml/kg) and xylazine (10 ml/kg). Fur on the top of the head was removed using clippers and a depilatory cream. The tail vein was catheterized with a 24-gauge catheter (Surflo®, Terumo) for intravenous (IV) administration.

When brain samples were needed for histology, animals were sacrificed via transcardial perfusion with saline followed by 10% formalin. Brains were fixed in 10% formalin for 48 h before processing. When brain samples were not taken, animals under deep anesthesia were euthanized with a lethal injection of pentobarbital (Euthasol®, 0.1 mL IV).

**Figure 1:**
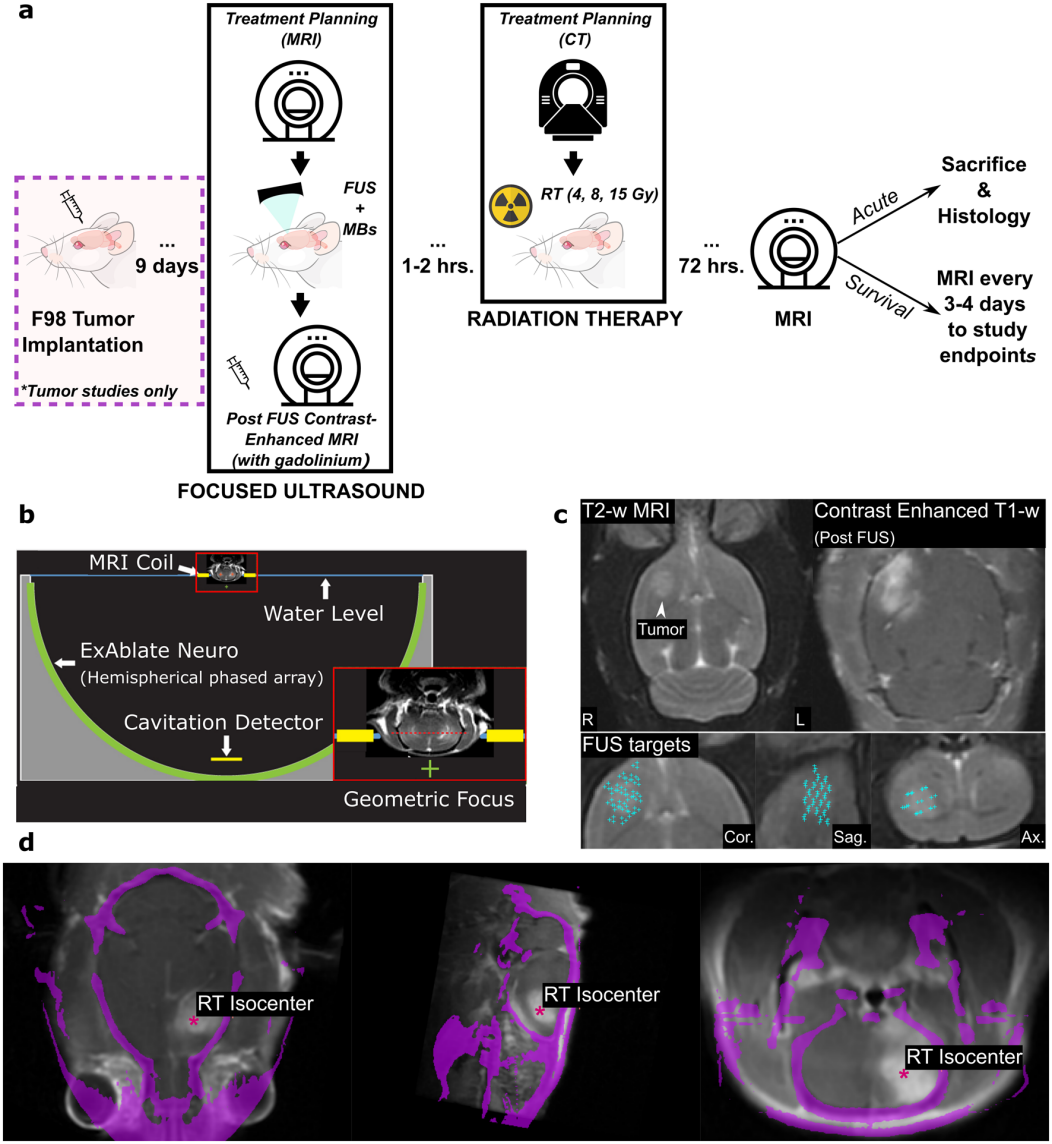
Diagrams showing the experimental methods used in the study. (**a**) An outline of the experimental timeline used across experimental groups, including healthy and tumor rats, and survival and acute studies. [This schematic has been designed using images from Adobe Stock Images and Flaticon.com.] (**b**) A schematic representation of the ExAblate Neuro System, adapted for preclinical use in the rat brain. (**c**) Example MRIs describing volumetric ultrasound targeting of a tumor in the striatum using T2-weighted MRI and showing BBB opening on contrast enhance T1-weighted MRI after FUS. (**d**) Co-localization skull (pink) from CT images acquired for RT treatment planning and MRIs acquired during FUS treatments for RT isocenter selection.

An outline of the experimental timeline is shown in Fig. 1a.

### Focused ultrasound

A Magnetic Resonance Imaging (MRI)-guided FUS device (ExAblate Neuro 220 kHz, InSightec), that has previously been used clinically for BBB disruption^25–27^, was used for ultrasound sonications. This device consists of a 30 cm-diameter, 1024-element, steerable, hemispherical transducer array, a cavitation monitoring system, and a water system for cooling, degassing, and circulation. Details on the adaptation of this device for preclinical studies and targeting have been described by McDannold et al. for BBB opening^28^. A schematic of the device used for FUS sonications is shown in Fig. 1b. In brief, in each subject, 20 overlapping targets were sonicated in a volume that covered most of the striatum (healthy rats) or that covered the tumor and a surrounding margin in the striatum, at a central axial plane. An example of selected FUS targets in a rat with a tumor is shown in Fig. 1c. The targets had a 3 mm center-to-center spacing and were arranged to conform to the shape of the striatum in the rat brain^28^. Over each of the 20 targets, the focus was steered to three sub-sonication targets 0.5 mm apart. This micro-steering aimed to slightly enlarge the targeted region and yield more homogeneous results.

Prior to FUS sonications, 3D T2*-weighted MRIs were manually registered to a template using software developed in-house. Baseline sonications (total duration: 35 s) were first delivered without MBs for comparison to treatment sonications and to confirm a lack of cavitation activity. Sonications were then repeated with MBs for the actual treatment (total duration: 180 s). MBs (Definity®, Lantheus Medical Imaging) were administered at the start of the sonication as a bolus injection through the tail vein at twice the approved clinical dose for ultrasound imaging, 20 µl/kg (~ 2.4 × 10^8^ bubbles/kg). To facilitate the injections, the agent was diluted 10:1 in saline and was followed by a 200 µl injection of saline. FUS was delivered to the 20 target locations in the striatum over two volumetric sonications with 10 targets. Each volumetric sonication consisted of 5 ms bursts applied sequentially to the 10 targets with a pulse repetition frequency of 1.1 Hz interleaved over 180 s. A delay of at least three minutes ensured that most of the microbubbles had cleared before the start of the second sonication.

Acoustic powers were modified using a real-time feedback controller, based on the analysis of acoustic emissions from cavitating MBs^29–31^. The starting acoustic power for sonications was 0.16 W, and the power was not allowed to exceed 2.58 W. Based on calibrations performed with a 4 mm-diameter omni-directional needle hydrophone (Reson TC4038, Teledyne), these powers corresponded to estimated peak negative pressures in the range 102–444 kPa in water. The Reson TC4038 was calibrated for low frequencies by measuring the acoustic pressures, over a range of driving voltages, at the focus of an in-house fabricated, 274.3 kHz focused transducer of known calibration. It has previously been reported that transmission through the rat skull is in the range of 76–86% at a comparable frequency of 268 kHz^32^. Passive monitoring of cavitation signals was performed using two receivers/hydrophones. The first was integrated into the ExAblate device and was resonant at the subharmonic frequency (115 kHz). The second was an in-house fabricated, elliptical (5 × 3 cm), air-backed receiver, with a resonant frequency of 660 kHz. A proportional feedback controller was used to independently modulate the power at each target during the exposures based on the strength of the second and third harmonic emissions at 440 and 660 kHz. The acoustic power was ramped until these signals reached a harmonic goal of 13–15 dB above the noise floor. The controller was designed to maintain harmonic emissions within this band for the first 30 s of the sonication, after which the power was set to the average power at which the harmonic goal was achieved during that time. If either wideband, subharmonic, or ultraharmonic (550 kHz) emissions were detected at any point during the sonication, the power was decreased by 15% and was not allowed to exceed that value for the remainder of the sonication. Detailed methods on this method of feedback control and methos for acoustic emissions acquisition with this set up have previously been described by Sun et al.^30^ and McDannold et al.^28^.

### Radiation therapy

Following FUS sonications, within 1–2 h, the target tissue was irradiated using the Small Animal Radiation Research Platform (SARRP; XStrahl). Computed Tomography (CT) images acquired by the SARRP system for treatment planning were manually registered with 3-plane T2-weighted MRIs and Contrast-Enhanced T1-weighted MRIs in 3D Slicer^33^. This step allowed the confirmation of co-localization of the FUS and RT targets. An example of CT-MRI registration and RT isocenter selection is shown in Fig. 1d.

The SARRP was operated at 220 kVp with a 0.15 mm Cu filter. Targets were placed at isocenter and irradiated using RT doses of either 4, 8 or 15 Gy using a 3 × 3 mm collimator. The subjects were placed prone, and two fields were administered, one at 0° and one at 180°. The dose-rate at isocenter was 222 cGy/min (0°) and 174 cGy/min (180°). The SARRP treatment planning system was commissioned using measurements with an ADCL-traceable ion chamber, following the procedure of AAPM TG-61. The dose-rate at isocenter is confirmed monthly by ion chamber measurements in solid water.

### Magnetic resonance imaging

MRI was used for treatment planning, to evaluate BBB disruption, to monitor therapeutic outcomes and tumor progression, and to detect tissue damage.

The ExAblate Neuro device was integrated into a clinical MRI machine (Signa Premiere 3 T, GE Healthcare). An in-house constructed, rectangular receive-only surface coil (dimensions: 5 × 6 cm) was integrated with the system for imaging during the brain the experiments^28^.

Prior to FUS sonications, a T2-weighted fast spin echo (FSE) sequence was acquired to visualize the brain and tumor, and a 3D T2*-weighted Susceptibility Weight Angiography (SWAN) sequence was acquired for treatment planning. After sonication, T2-weighted images were acquired to assess edema, and T2*-weighted images were collected to assess vascular damage. A T1-weighted FSE sequence was acquired before and after administration of MRI contrast (Gadavist, Bayer HealthCare Pharmaceuticals, 0.125 mmol/kg) to assess BBB opening and confirm FUS-mediated bioeffects. Post-FUS MRIs were registered to Computed Tomography (CT) images in 3D Slicer^33^ for treatment planning prior to irradiation.

For all animals in the study, follow-up MRIs (T2-weighted, Susceptibility-weighted imaging (SWI), contrast-enhanced T1-weighted) were acquired at 72 h post-treatment using a small-animal MRI (BioSpin 7 T, Bruker). A 15 mm inner-diameter, receive-only surface coil (Bruker) was used to enhance signals in the brain.

### Healthy rats

FUS and RT were used to target the right striatum in 19 male, healthy Sprague Dawley rats. The animals were divided into five treatment groups as follows: (1) FUS only (n = 5), (2) 8 Gy only (n = 3), (3) 15 Gy only (n = 3), FUS + 15 Gy (n = 4), and FUS + 8 Gy (n = 4). FUS and RT were applied as described previously. The animals were monitored for 21 days after treatment. 7T T2-weighted MRI was acquired at days 3, 7, 10, 14, 17 and 21 to assess gross morphological and anatomical changes. On day 21, rats were sacrificed via transcardial perfusion. Brains were harvested and stained for hematoxylin and eosin (H&E).

### Tumor rats

F98 tumor cells were implanted into the right striatum of 65 male Fischer rats. Wild-type F98 cells (passage number five, CRL-2397, American Type Culture Collection) were cultured in high glucose Dulbecco’s Modified Eagle Medium (DMEM, 1×) supplemented with 10% Fetal Bovine Serum (FBS) and 5% Penicillin Streptomycin (P/S) in a humidified incubator with 5% CO_2_ at 37 °C. In anesthetized Fischer rats, the dorsal surface of the skull was sterilized with an iodine swab. A 1 cm linear skin incision was placed over the bregma, and a 1 mm burr hole was drilled into the skull approximately 1 mm superior to and 3 mm lateral to the bregma. A 4 μL cell suspension (4.5 × 10^6^ cells/ml) was injected into the caudate putamen 3.5 mm from the dura surface using a 26-gauge, 10 μL airtight syringe (Hamilton). The cell suspension was injected at a rate of 0.9 µL/min using a digital syringe pump (Nanomite, Harvard Apparatus). Following the injection, the needle was allowed to sit for 3 min, before being slowly withdrawn over 3 min. Animal behavior was monitored daily after surgery and sutures were removed after 5 days.

Tumor rats were treated as either part of a survival or acute study, as described below. An experimental timeline for the survival and acute tumor studies is shown in Fig. 1a. We added a lower radiation dose of 4 Gy to the tumor study based on the outcomes of the healthy brain study where lesions were evident at 8 Gy.

### Survival study

55 rats were included in the F98 survival study to assess the effects of FUS ± RT (4, 8, and 15 Gy) on tumor growth and survival. These animals were divided into 10 treatment groups, with 4–8 animals per group, as outlined in Supplemental Table 1. FUS_BBB_ indicates the use of ultrasound parameters that have previously been used for BBB opening with the ExAblate System^28^. In those rats, the MB dose was reduced to 10 μl/kg and the harmonic goal used in the feedback control was reduced by 3 dB to 10–12 dB above the noise floor; other parameters were the same. The same proportional controller described for the FUS parameters, based on the strength of harmonic emissions at the second and third harmonics, was used for the FUS_BBB_ group.

**Table 1:** Tumor doubling times for animals in the control, Focused Ultrasound (FUS) only, Radiation Therapy (RT) only, and FUS + RT Treatment Groups.

	Tumor Doubling Time, T_D_—mean (standard deviation) days							
	Control	FUS	15 Gy	FUS + 15 Gy	8 Gy	FUS + 8 Gy	4 Gy	FUS + 4 Gy
HIV	2.93 (0.08)	2.46^§^ (0.36)	3.73 (0.47)	4.08^§^ (0.19)	3.50^§^ (0.42)	3.52^§^ (0.33)	2.94 (0.06)	3.16 (0.26)
Core	1.88 (0.01)	1.60 (0.26)	2.61^§^ (0.32)	2.74^§^ (0.16)	2.34^§^ (0.28)	2.37^§^ (0.24)	1.87 (0.06)	2.08^§^* (0.13)

*: *p* < 0.05 compared with RT only group.

^§^: *p* < 0.05 compared with Control group.

Following treatment on Day 9 after tumor implantation, 7T T2-weighted MRI was acquired every 3–4 days, until study endpoints, to assess anatomical changes and tumor progression. To quantify tumor progression, the tumor cores, and Hyperintense Volumes (HIVs) surrounding the tumors were manually segmented in 3D Slicer.

### Study endpoints

Healthy rats were sacrificed 21 days after FUS. Tumor-bearing animals were euthanized when they showed impaired activity, weight loss more than 20% of their body weight in one week, or a tumor core diameter above 1 cm.

### Acute study

10 rats were included in the F98 acute study to assess the effect of FUS + RT on apoptosis and ceramide levels at the 72 h time point. These animals were divided into three groups: (1) FUS only (n = 4), (2) 4 Gy only (n = 3), and (3) FUS + 4 Gy (n = 3). Treatment was delivered 9 days after tumor implantation. 72 h after the treatment (12 days after tumor implantation), the brains were imaged using 7 T T2-weighted MRI. The animals were then sacrificed via transcardial perfusion, and the brains were harvested and stained with H&E. The brains were also stained for apoptosis, using a Terminal deoxynucleotidyl transferase dUTP nick end labeling (TUNEL) assay, and for ceramide, and markers of the innate and adaptive immune system, as described below.

### Histology

Samples were paraffin embedded, sectioned, and stained for H&E at the Dana Farber/Harvard Cancer Center Rodent Histopathology Core. 5 µm sections were obtained 250 µm apart.

In each brain, 5 µm sections 500  µm apart were processed for TUNEL staining and co-stained using immunohistochemistry to detect CD31 (blood vessels) and ceramide. TUNEL staining was performed using a TUNEL Assay Kit—HRP-DAB (ab206386, Abcam). For CD31, a 1:200 dilution of ab1822981 EPR17259 (Abcam) was used as the primary antibody, while a 1:500 dilution of Alexa Fluor® 568 (ab175471, Abcam) was used as the secondary antibody. For ceramide staining, a 1:20 dilution of a monoclonal anti-ceramide antibody (C8104-50TST, Sigma-Aldrich) was used as the primary antibody, while a 1:200 dilution of Alexa Fluor® 647 (ab150115, Abcam) was used as the secondary antibody. Heat-mediated antigen retrieval of paraffin-embedded sections was performed in a pressure cooker using ab93684 (Abcam; 100 × Tris–EDTA Buffer, pH 9.0).

5 µm sections 500 µm apart were co-stained using immunofluorescence to detect microglia/macrophages (Ionized calcium binding adaptor molecule—Iba1) and macrophages (F4/80), and generalized T cells (CD3) and cytotoxic T cells (CD8). For Iba1 and F4/80, 1:200 dilutions of an anti-Iba1 antibody, ab5076 (Abcam), and an anti-F4/80 antibody, ab300421 (Abcam) were used as the primary antibodies, while 1:500 dilutions of Alexa Fluor® 568 (ab175471, Abcam) and Alexa Fluor® 647 (ab150115, Abcam) were used as the secondary antibodies. Heat-mediated antigen retrieval of paraffin-embedded sections was performed in a pressure cooker using ab93684 (Abcam; 100 × Tris–EDTA Buffer, pH 9.0). For CD3 and CD8, 1:200 dilutions of an anti-CD3 antibody, ab16669 (Abcam), and an anti-CD8 antibody, ab33786 (Abcam) were used as the primary antibodies, while a 1:500 dilution of Alexa Fluor® 568 (ab175471, Abcam) and a 1:200 dilution of Alexa Fluor® 647 (ab150115, Abcam) were used as the secondary antibodies. Heat-mediated antigen retrieval of paraffin-embedded sections was performed in a pressure cooker using ab93678 (Abcam; 100 × Citrate Buffer, pH 6.0).

For all immunofluorescence protocols, the primary antibodies were incubated overnight at 4 °C and the secondary antibody were incubated for 2 h at room temperature in the dark. 4% Bovine Serum Albumin (BSA; A2153-50G, Sigma-Aldrich) was used as a blocking solution and to dilute the primary and secondary antibodies. 4′,6-diamidino-2-phenylindole (DAPI, R37606, Invitrogen) was used as a counterstain to visualize cell nuclei.

Following staining, the slides were digitized using a VS-120 Slide Scanner (Olympus; Neurobiology Imaging Facility, Harvard Medical School) for brightfield images or a ZEISS Z1 Observer Microscope (Zeiss) for fluorescent images at 20× magnification. FIJI^34^ was used to quantify the levels of the different markers in the sample tissue. Digitized TUNEL stained slides were manually thresholded in FIJI to determine the TUNEL positive areas in the tumor and the striatum. TUNEL positive areas were normalized to the total area of the region of interest (ROI). Similarly, CD31 and Ceramide-stained slides were manually thresholded in FIJI. Since the effect of FUS + RT is expected to be concentrated in the blood vessels^16^, the ratio of Ceramide to CD31 in the tumor was calculated. Manual thresholding to remove background noise was performed at the discretion of a single researcher who was blinded to the experimental parameters.

For the Iba1 and F4/80 channel measurements, areas were measured in the tumor ROI and a periphery ROI. The periphery ROI was defined to be the tumor ROI expanded outwards by 250 pixels (1 pixel = 0.512 µm), minus the original tumor ROI. Any large vessels or artifacts inside both ROIs were excluded from the analysis, as well as any necrotic cores inside of the tumor. All Iba1/F4/80 images were normalized through histogram matching amongst their respective channels and then thresholded using Otsu’s method^35^. The area of the thresholded cells was then calculated and normalized by the tumor ROI. The CD3/CD8 channel measurements were calculated in the same manner as the Iba1/F4/80 measurements, though due to concerns of background noise from the staining process, the number of cells rather than their area was measured to give an estimate of cells per unit area. To further account for noise, only cells that overlapped with regions of the thresholded DAPI images were included in these counts.

### Statistical analysis

All statistical analyses were performed in MATLAB, unless otherwise specified. A one-way ANOVA was post-hoc Least Squared Difference testing was used to assess differences between the groups at the *p* < 0.05 significance level. To assess effects on survival, Kaplan–Meier survival curves were plotted, and the Max-Combo statistical test, with great weighting on earlier events, was performed in R to detect statistically significant differences in survival^36^. The Max-Combo statistical test was chosen instead of the conventional Mantel-Cox Log-Rank test as it has been shown to be more robust for detecting differences in cases of non-proportional hazards, such as crossing survival curves^36,37^.

## Results

### Longitudinal effects in the healthy rat brain (FUS + RT—8 Gy and 15 Gy)

**Figure 2:**
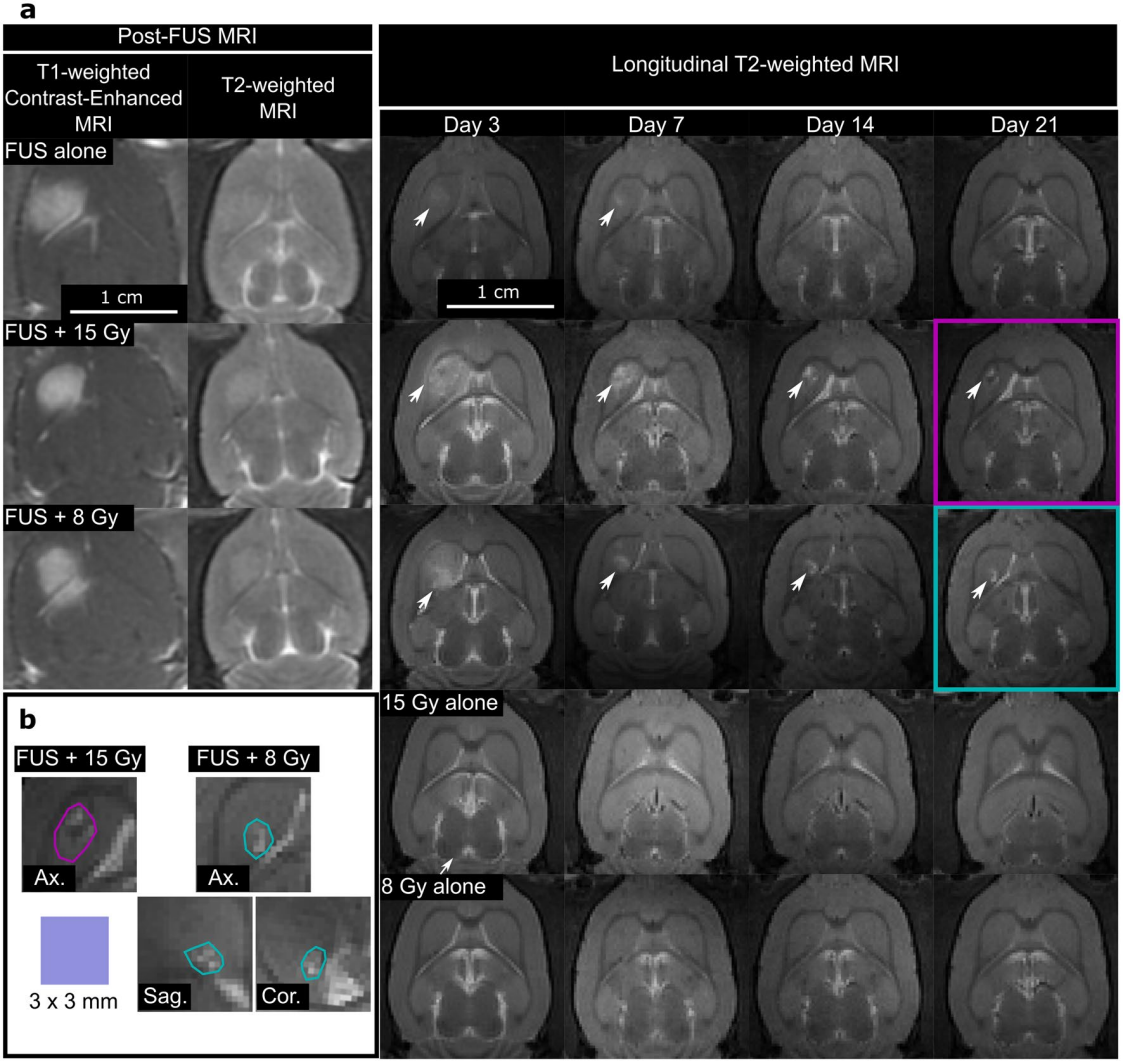
MRI acquired after FUS ± RT in healthy rats. (**a**) Shows representative T2-weighted images following treatment with RT alone, FUS alone, and FUS + RT over a period of 21 days. Representative contrast-enhanced T1-weighted images are shown for animals that received FUS. Post-FUS treatments show T1-weighted contrast enhancement and edema on T2-weighted images in all groups immediately after FUS. T2-weighted longitudinal MRI showed no observable effects in the RT alone groups, while a region of edema (white arrows), that resolved between day 10 and 17, was observed at the target of the FUS alone group. In the FUS + RT groups, edema and a lesion that persisted until day 21 was observed at both RT doses (white arrows). (**b**) Shows magnified versions of the observed lesions in the FUS + RT groups at day 21 post-treatment, including representative images in the axial, sagittal, and coronal planes.

Figure 2a shows representative MRI in healthy rats over 21 days following targeted treatment of the right striatum with RT alone (15 Gy; n = 3 and 8 Gy; n = 3), MB-mediated FUS alone (n = 5), and FUS + RT (15 Gy; n = 4 and 8 Gy; n = 4). In animals that received MB-mediated FUS, post-FUS T1-weighted contrast-enhanced MRI showed BBB opening (BBBO) in the targeted region of the brain. At the exposure parameters used, this corresponded to a region of edema observed on T2-weighted MRI. In 1/13 rats (FUS alone), minor T2* effects were observed on SWAN MRI.

In rats treated with RT alone, no gross morphological changes were observed on MRI over 21 days. With FUS alone, a hyperintense lesion was evident in the targeted volume after 3 days, but it resolved between day 10 and 17 after the treatment. In the FUS + RT groups, T2 hyperintense lesions qualitatively similar to those with FUS alone were observed on day 3, but they were more severe and persisted until animals were sacrificed on day 21. On day 7, these lesions were brighter than those in the FUS alone group. Over the next two weeks, the lesions shrank, and an enlargement of the adjacent ventricles was evident, indicating the removal of tissue. At the study endpoint, the rats showed no obvious signs of motor or neurological deficits. Representative images of segmented lesions at day 21 after FUS + RT in healthy brains is shown in Fig. 2b.

**Figure 3:**
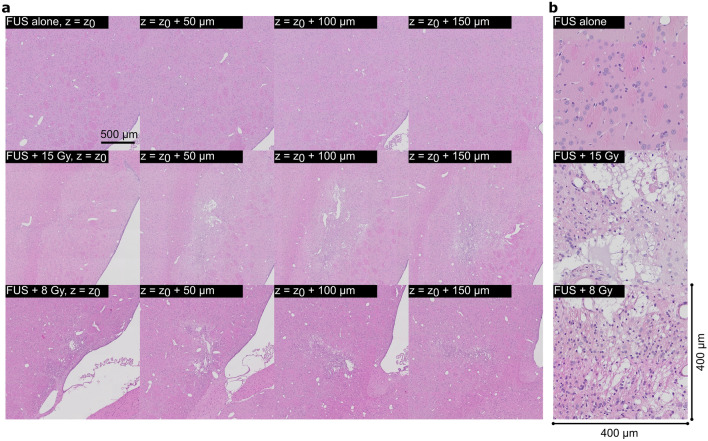
Representative, adjacent H&E slides, imaged at 20× magnification, for animals treated using FUS alone and FUS + RT (8 and 15 Gy). The targeted brain regions in animals treated with FUS + RT showed evidence of tissue damage, including tissue removal, scarring, and bleaching. (**a**) Shows adjacent, macroscopic 2 mm × 2 mm H&E images of the striatum that was treated. (**b**) Shows 400 µm × 400 µm close up representative images of the lesions.

Figure 3a shows adjacent, representative H&E-stained brain sections (20× magnification) in animals treated with FUS alone and FUS + RT at day 21 post-treatment in a 2 mm × 2 mm field-of-view. Figure 3b shows representative images of the lesions in a 400 µm × 400 µm field-of-view. In 4/5 animals treated with FUS alone, there was no evidence of tissue damage. In the remaining animal—the same animal where minor T2* effects were observed after the treatment—there was evidence of scarring at the treated location. In 4/4 animals treated with FUS + 15 Gy and 4/4 animals treated with FUS + 8 Gy, H&E staining showed evidence of tissue removal, scarring, and bleaching that was consistent with the lesions observed using MRI. No damage to the brain tissue was observed in animals treated with RT alone.

### The effect of FUS + RT on tumor growth and survival in a F98 glioma model

**Figure 4:**
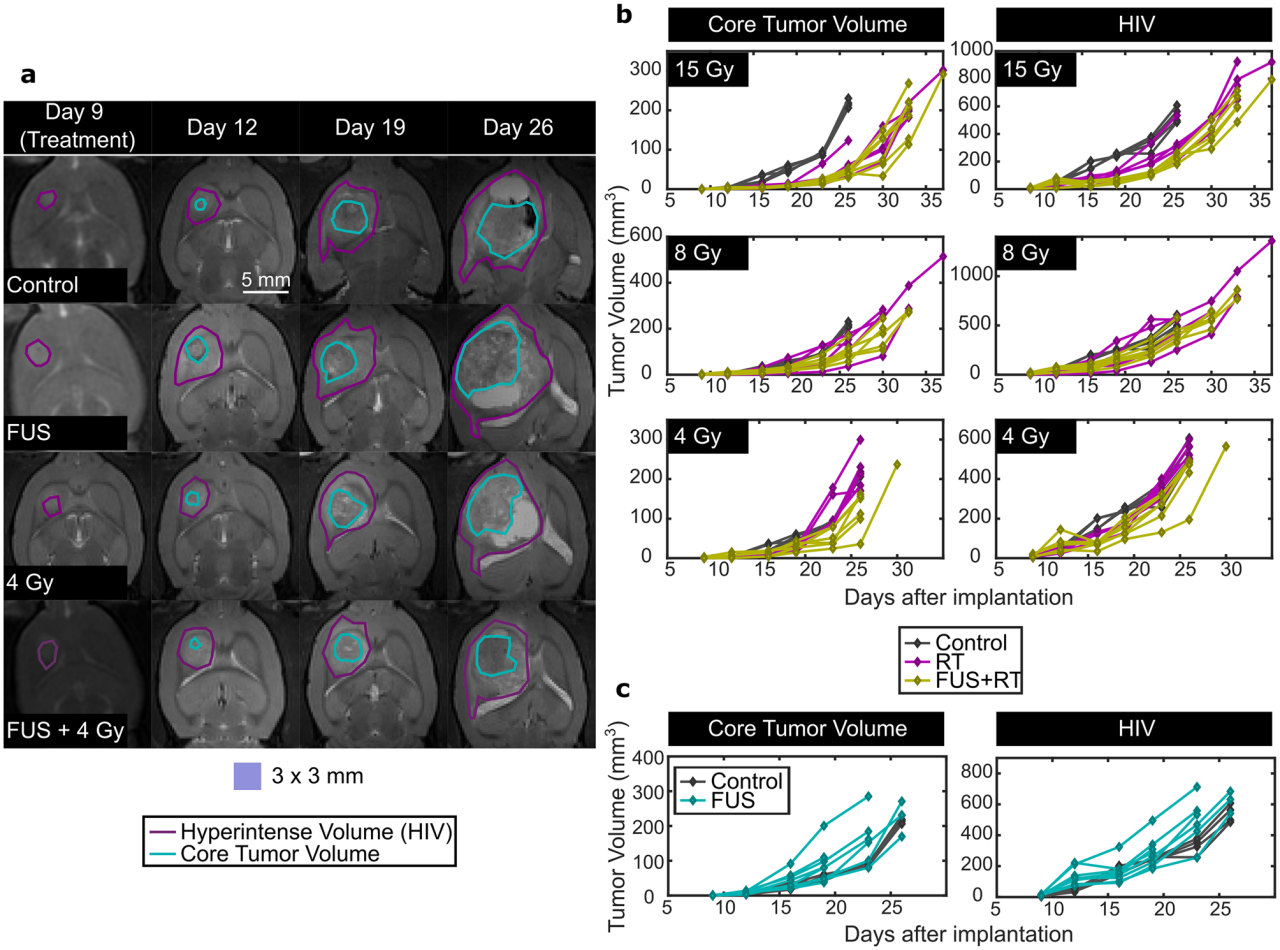
MRIs and plots demonstrating tumor progression. (**a**) Shows representative examples of T2-weighted images showing tumor growth. Segmentations of the core tumor and surrounding hyperintense volumes are shown for animals in the control, FUS only, 4 Gy only and FUS + 4 Gy treatment groups. (**b**) Shows plots of the growth of the tumor core and surrounding hyperintense region in the F98 survival study for the control, RT only, and FUS + RT groups. Each line represents an individual subject and plots are grouped by the RT dose. (**c**) Shows plots of the growth of the tumor core and surrounding hyperintense region for the control and FUS only groups. The FUS only plots are shown on separate graphs for figure clarity.

On the day of treatment, across all treatment groups, the initial size of the HIV was 10.4 ± 5.6 mm^3^ (mean ± standard deviation), and a necrotic tumor core could not be identified using MRI. Example segmentations of the HIV and tumor core are shown in Fig. 4a. A delay in tumor growth was evident in many of the animals that received FUS and RT. Figure 4b shows tumor growth curves grouped by RT dose for control, FUS only, RT only and FUS + RT animals. The curves shown in Fig. 4b were each fitted to exponential functions, and the resulting tumor doubling times (T_D_, i.e. the time taken for a tumor to double its size) are shown in Table 1. The tumor growth curves for the FUS only group are shown in Fig. 4c. FUS only reduced T_D_ compared to the control group. This reduction was not statistically significant for the tumor core (*p* = 0.064), but was statistically significant for the HIV (*p* = 0.028). T_D_ was significantly longer (*p* < 0.05) for animals that received RT at 8 and 15 Gy alone compared to control animals. T_D_ for both the core and the HIV increased with the addition of FUS at these doses, but the increases were not significant compared to animals that received RT alone. Only the FUS + 4 Gy animals showed a small but significant (*p* = 0.003) increase in the T_D_ of the tumor core when compared to animals that received 4 Gy only.

**Figure 5:**
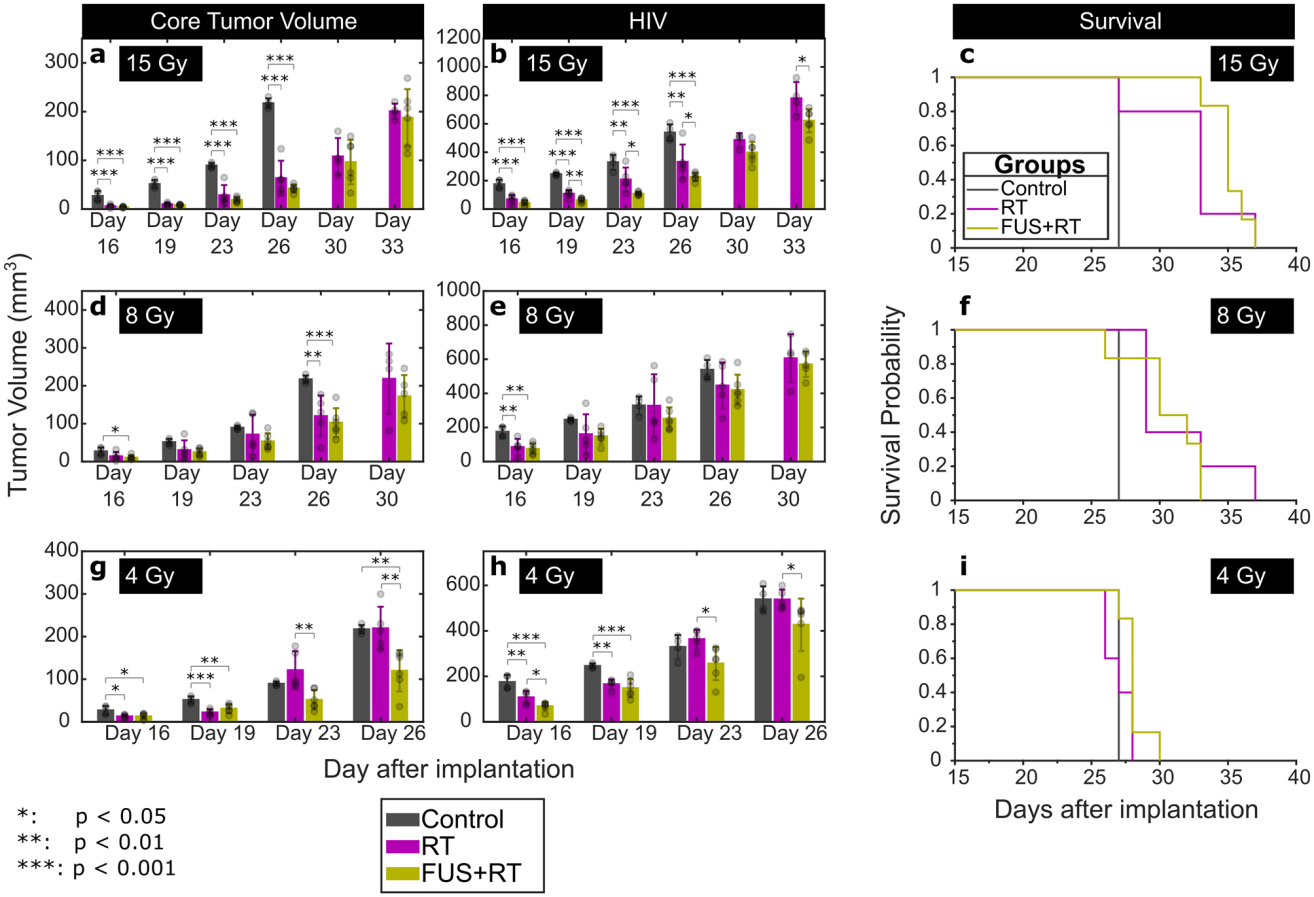
Tumor volumes and survival plots for animals receiving no treatment, RT and FUS + RT in the survival study, grouped by radiation dose (15, 8, and 4 Gy). The histograms show the means and standard deviations for each group, as well as individual data points.

In Fig. 5, tumor burdens starting at day 16 after tumor implation are shown at each point of measurement to better delineate the differences among groups for animals receiving no treatment, RT, and FUS + RT. The purpose of this analysis was to assess the therapeutic efficacy of the combined treatment compared to RT alone. Data before day 16 were not analyzed as edema following FUS treatments made it difficult to identify the HIV, leading to the overestimation of these values. Kaplan–Meier Survival Plots are also shown for each RT dose.

At a RT dose of 15 Gy, both core volume and HIV was significantly reduced by RT alone compared to the control group from Day 16 to Day 26 (Fig. 4b). No further significant reduction in the core volume was obsered using FUS + RT. However, significant reductions in the HIV using FUS + RT compared with RT alone were observed at Day 19 (*p* = 0.002), Day 23 (*p* = 0.021), Day 26 (*p* = 0.044) and Day 33 (*p* = 0.033). The median survival for animals in the control group was 27 days (range: 27–27 days) compared with 33 days (range: 27–37 days) and 35 days (range: 33–37 days) in the 15 Gy and FUS + 15 Gy groups respectively (Fig. 5c). Kaplan–Meier and Max-Combo Analysis indicated that there was a significant difference in survival between control animals and those treated with 15 Gy alone (*p* = 0.012) and between control animals and those treated with FUS + 15 Gy (*p* = 0.001). The improvement in survival from the 15 Gy alone group to the FUS + 15 Gy group was not statistically significant (*p* = 0.059).

At a RT dose of 8 Gy, the tumor response to RT alone was highly variable. A one-way ANOVA did not identify any significant differences between the RT only and FUS + RT groups (Fig. 5d–e). For the core tumor volume, on Day 16, the core tumor volume was significantly reduced with FUS + RT compared with controls (*p* = 0.016). At the same time, RT alone did not yield a significant difference from the controls (*p* = 0.055), and there was no difference between RT alone and FUS + RT (*p* = 0.530). Figure 5f shows the Kaplan–Meier Survival Plots for these groups. Compared with the median survival of 27 days for the control group, animals treated with 8 Gy alone had a median survival of 29 days (range: 29–37 days) and animals treated with FUS + 8 Gy had a median survival of 30 days (range: 26–33 days). These were both significantly different from the control group (8 Gy: *p* = 0.002; FUS + 8 Gy: *p* = 0.031), but not from each other (*p* = 0.426).

At a RT dose of 4 Gy, statistically significiant differences were observed between the tumor volumes in rats treated with RT alone and FUS + RT. At this dose, the response to RT alone was mimimal, with no significant reductions in tumor volumes compared with the controls at Day 19. At Day 23 and Day 26, the mean core tumor volume was reduced by 57.4% (*p* = 0.002) and 45.4% (*p* = 0.002) respectively, using FUS + RT compared with RT alone. Significant differences in the HIV between the 4 Gy only and FUS + 4 Gy groups were also observed at Day 16 (*p* = 0.025), Day 23 (*p* = 0.011), and Day 26 (*p* = 0.048). Compared with the median survival of 27 days for the control group, animals treated with 4 Gy alone had a median survival of 27 days (range: 26–28 days) and animals treated with FUS + 4 Gy had a median survival of 28 days (range: 27–30 days). The former was not statistically significant from the survival in the control group (*p* = 0.500), while the latter was (*p* = 0.007). The improvement in survival from the 4 Gy alone group to the FUS + 4 Gy group was also statistically significant (*p* = 0.041).

### FUS only

**Figure 6:**
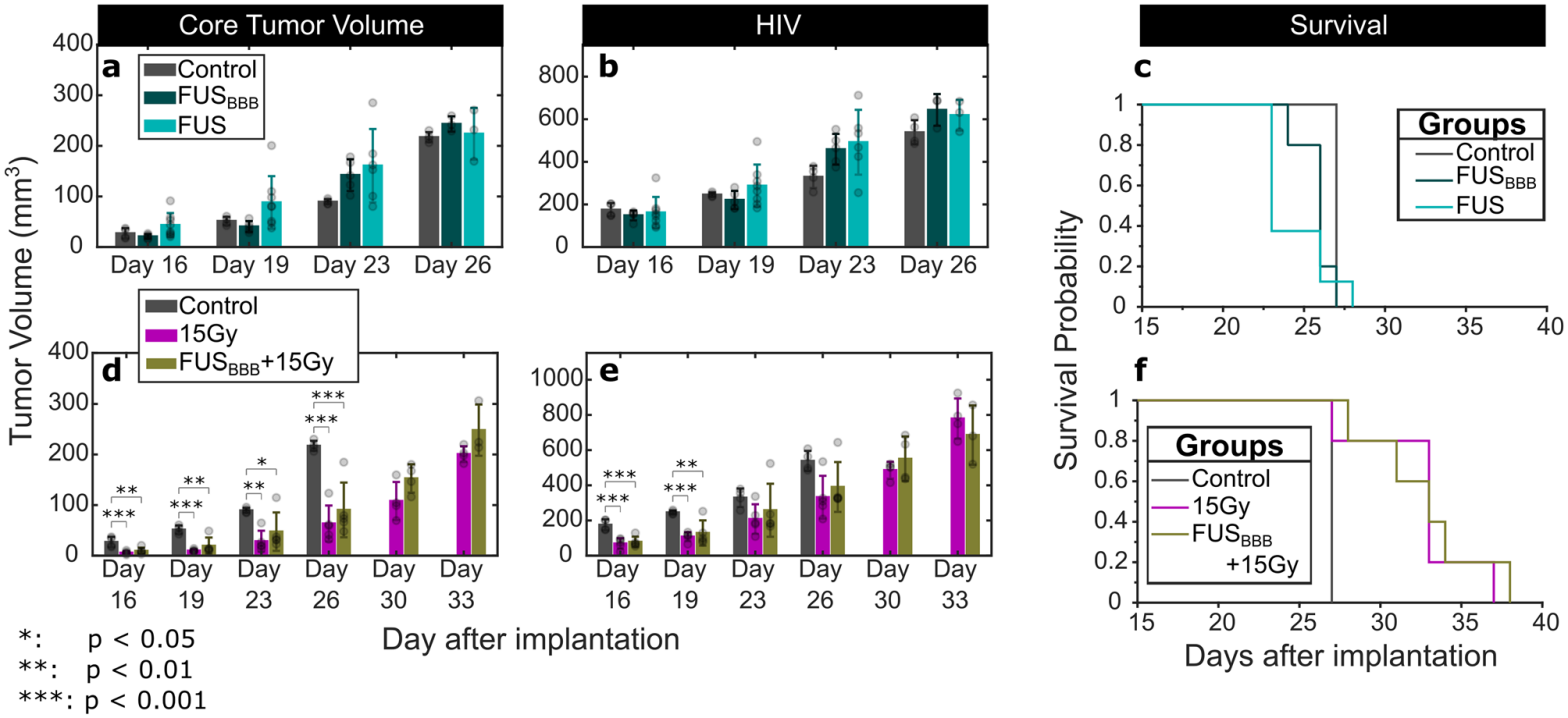
Tumor growth and survival for animals treated with FUS only and FUS_BBB_ parameters. (**a**–**c**): Tumor growth and survival for animals in the control, FUSBBB and FUS groups. The core tumor volumes and hyperintense tumor volumes for these groups are shown in (**a**) and (**b**) respectively. (**c**) Shows the Kaplan–Meier survival plots for animals in these groups. (**d**–**f**): Tumor growth and survival for animals in the control, 15 Gy only and FUS_BBB_ + 15 Gy groups.

Figure 6a-c show the tumor growth and survival for controls (no treatment), FUS_BBB_ alone and FUS alone. Mean tumor volumes were greater in both treatment groups than the control groups, but a one-way ANOVA indicated no statistically significant differences among these groups at different time points, for both the core volumes (Day 16: *p* = 0.095; Day 19: *p* = 0.091; Day 23: *p* = 0.124; Day 26: *p* = 0.523) and HIVs (Day 16: *p* = 0.770; Day 19: *p* = 0.296; Day 23: *p* = 0.103; Day 26: *p* = 0.159). The median survival for animals in the control group was 27 days (range: 27–27 days) compared with 26 days (range: 24–27 days) and 23 days (range: 23–28 days) in the FUS_BBB_ and FUS-only groups respectively. However, there was a significant difference in survival between control animals and those treated with FUS_BBB_ (*p* = 0.015) and those treated with FUS (*p* = 0.016). No difference was observed between the FUS and FUS_BBB_ groups (*p* = 0.757).

### FUS_BBB_ + RT

Figure 6d-f show the tumor growth and survival for controls (no treatment), 15 Gy only and FUS_BBB_ + 15 Gy. A one-way ANOVA and post-hoc testing showed statistically significant differences between control animals and animals receiving a radiation dose of 15 Gy in both the core tumor volumes (Days 16, 19, 23 and 26; Fig. 6d) and the HIV (Days 16 and 19; Fig. 4b). However, no statistically significant changes in tumor burden were observed between the 15 Gy only and FUS_BBB_ + 15 Gy groups at the *p* < 0.05 level. Figure 6f shows the Kaplan–Meier survival curves for the three groups. The median survival for both the 15 Gy and FUS_BBB_ + 15 Gy groups was 33 days (27–37 days, 15 Gy; 28–38 days, FUS_BBB_ + 15 Gy). While the change in survival was statistically significant compared with the control group (*p* = 0.012, Control vs. 15 Gy; *p* = 0.002, Control vs. FUS_BBB_ + 15 Gy), no difference was observed between the 15 Gy and FUS_BBB_ + 15 Gy groups (*p* = 0.416).

### Histological findings in a F98 glioma model at 72 h

**Figure 7:**
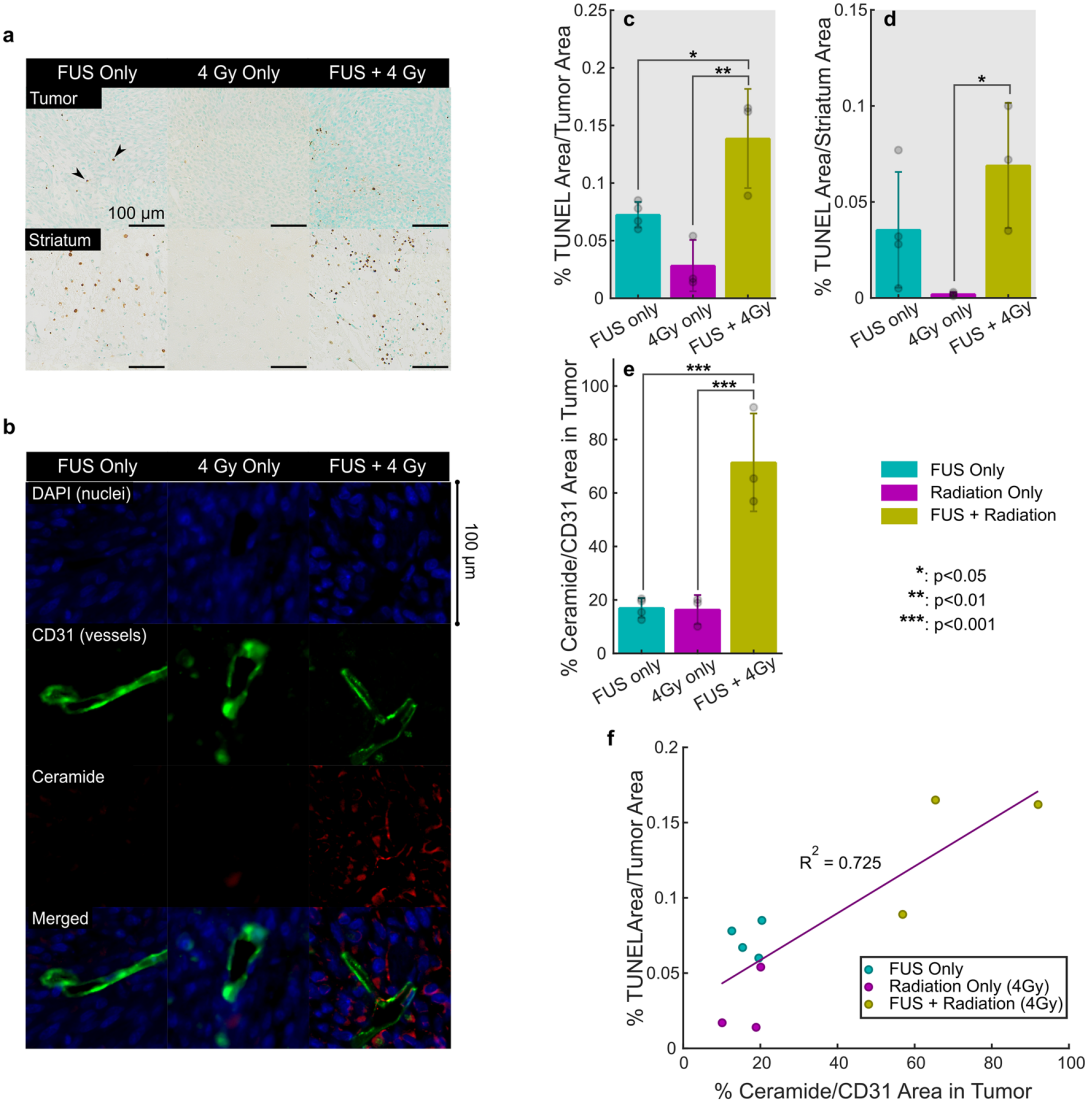
Histological Findings in a F98 Glioma Model at 72 h. (**a**) Shows representative images of TUNEL (apoptosis) staining in tumors and the surrounding brain tissue at 72 h. after treatment in animals treated with FUS only, 4 Gy Only and FUS + 4 Gy. (**b**) Shows representative images of CD31 (blood vessels) and ceramide staining, showing increased vessel-associated ceramide in tumors treated with FUS + RT at 72 h. after treatment. (**c**–**f**) Show quantitative measures of TUNEL and Ceramide: (**c**) Area of TUNEL (apoptosis) positive cells in tumors normalized to tumor area. (**d**) Area of TUNEL positive cells in the surrounding striatum normalized to tumor area. (**e**) Relative ceramide to CD31 (blood vessels) area in tumors. (**f**) Relationship between TUNEL (apoptosis) and Ceramide.

Figure 7a shows representative TUNEL staining at 72 h for animals treated with FUS only, 4 Gy only and FUS + 4 Gy. Qualitatively, these images show increased TUNEL positive apoptotic cells in both the tumor and the striatum with FUS + 4 Gy. FUS only images showed increased levels of apoptosis compared with 4 Gy alone.

Figure 7b shows representative immunofluorescent staining at 72 h for animals treated with FUS only, 4 Gy only and FUS + 4 Gy. Qualitatively, these images show increased ceramide with the combined (FUS + 4 Gy) treatment than with either FUS or 4 Gy alone.

The results shown qualitatively in Fig. 7a–b are represented quantitatively in Fig. 7c–f. TUNEL staining at 72 h showed that FUS + 4 Gy led to a 93% (*p* = 0.015) and 396% (p = 0.002) increase in the apoptotic area in tumors compared with FUS and 4 Gy alone respectively (Fig. 7c). In the striatum (Fig. 7d), no statistically significant increase was observed with the combined treatment relative to FUS alone, but a significant increase of 3350% was observed compared with RT alone (*p* = 0.017). In Fig. 7e, immunofluorescent staining showed that FUS + RT led to a 320% (*p* = 2.5 × 10^–4^) and 336% (*p* = 3.6 × 10^–4^) increase in relative ceramide to CD31 (blood vessel) area in tumors compared with FUS and 4 Gy alone respectively. Figure 7f shows plots the percent TUNEL positive area plotted against the relative ceramide to CD31 area in the tumors. The amount of ceramide showed a good correlation with the TUNEL positive area in the tumors (R^2^ = 0.725).

No statistically significant changes in innate or adaptive immune markers were observed among the treatment groups (Supplemental Fig. 1). However, the average number of cytotoxic T-cells per unit area within the tumor with FUS + RT was increased by 198 and 86%, compared with FUS only and RT only respectively (ANOVA: *p* = 0.058).

## Discussion

In this paper, we investigated the effects of MB-mediated FUS + RT in the brains of healthy rats and in rats with F98 gliomas. In the healthy brain, FUS was combined with RT doses of 15 and 8 Gy. The results presented in this study show that at these doses, FUS + RT led to the formation of an ablative lesion in the treated location that could be identified using T2-weighted MRI within 14 days after treatment (Fig. 2) without neurological deficits. When animals were sacrificed 21 days after treatment, evidence of tissue clearance and scarring was seen on H&E-stained histology sections (Fig. 3). In contrast, ablative effects were not observed with either FUS or RT alone. FUS + RT may be a useful tool for ablative neurosurgery, including in neuro-oncology and the treatment of other neurological disorders. Providing that the size of the FUS focal volume can be finely tailored, this treatment may play a role in decreasing the RT dose currently used in RT-mediated neurosurgical approaches. For example, Gamma Knife Functional Radiosurgery for the treatment of Essential Tremor, typically uses marginal RT doses in the range 60–70 Gy^22^. Combining RT with FUS may be an effective means of reducing the RT dose required for Radiosurgery in such therapies. Furthermore, the lesion developed rapidly, with tissue removal evident one week after treatment. With Gamma Knife, effects are often not observed for over a month. However, more robust investigations of FUS + RT ablation are required to understand the effect observed here and to determine appropriate clinical targets.

In the F98 glioma model, the effect of combining FUS and 15 Gy RT on tumor growth and survival was mixed. At the highest RT dose investigated in this work, RT alone significantly reduced tumor burden and prolonged survival compared with animals that received no treatment. No further statistically significant reduction in core tumor volume or increase in survival was observed with the combined treatment. However, the HIV surrounding the tumor core was shown to be reduced using the combined treatments (Figs. 4b, 5b) between Day 19 and Day 26 after tumor implantation (Day 10–Day 17 after treatment). The role of cerebral edema and inflammation in the clinical representation of brain tumors is not fully understood, but it can lead to increased intracranial pressure and brain herniation, which can potentially be fatal^38^. While the impact of reducing the HIV in an aggressive cancer like GBM is minimal, this finding justifies further investigation into the potential anti-inflammatory response following FUS + RT.

At a RT dose of 8 Gy, RT alone led to variable responses in tumor volume compared to the control group (Figs. 4b, 5d–e) and increased survival. While the mean tumor volume was reduced across different time points used for measurements (Fig. 5d–e), the high inter-subject variability limited statistical significance. Combining 8 Gy RT with FUS did not significantly improve the performance of RT alone. It is possible that the middle RT dose of 8 Gy is on the boundary of the dose alone that is needed to be effective for improvement in a F98 glioma model. Previous studies using RT to treat F98 gliomas have used doses in excess of 15 Gy at the tumor location^39,40^.

A RT dose 4 Gy alone had limited impact on tumor growth and survival. Although there was a reduction in the core and hyperintense tumor volumes at the earliest time points after treatment (Fig. 5g–h), this was the only dose tested in this study that did not significantly increase the tumor doubling time compared to animals receiving no treatment (Table 1). This was the only dose tested where combining FUS and RT led to significantly reduced core tumor volumes compared with RT alone (Fig. 5g). Still, the survival benefit of the combined treatment was limited. The combined treatment showed a small, but significant improvement in survival in the FUS + 4 Gy group (median survival: 28 days) compared with the 4 Gy only group (median survival: 27 days, *p* = 0.041). There was no change observed between the control group (median survival: 27 days) and the 4 Gy only group, but the improvement compared to controls with the combined treatment was significant (*p* = 0.007). These data indicate that combining FUS and RT may be most effective at low RT doses where RT alone has limited therapeutic benefits. Further, 72 h following the treatment, combined FUS and RT at was shown to increase the apoptotic area in F98 tumors 93% compared with FUS alone (*p* = 0.015) and 396% compared with RT alone (p = 0.002). In line with the results presented by El Kaffas et al.^16^ this corresponded with an increase in ceramide of 320% compared with FUS alone (*p* = 2.5 × 10^–4^) and 336% compared with RT alone (*p* = 3.6 × 10^–4^). In the acute tumor study, a no-treatment control group would have been useful to be better able to interpret the impact of FUS alone and RT alone on tumor growth and should be included in future studies.

Two FUS only groups (FUS and FUS_BBB_) were included in the survival tumor study. While these treatments did not have a therapeutic advantage compared to the control group, results showed that both groups led to slightly increased tumor volumes, although these were not statistically significant (Fig. 6a and b). This corresponded to small, but statistically significant, decreases in median survival, from 27 days in the control group, to 26 days in the FUS_BBB_ group and 23 days in the FUS only group, suggesting that FUS-mediated BBB opening may have an adverse effect on tumors, in the absence of other therapeutic intervention. Previous studies in rodent models of glioma and breast-cancer brain metastases have shown marginal increases in tumor volumes in FUS only groups when compared to no-treatment controls, but these have not been linked to decreased survival^41,42^. In the case of this study, this observation may be due to limitations of the study design (i.e. small group size with no variance in the survival of the control group), or a physiological effect. As it pertains to the latter, FUS BBB opening has been linked to factors like increased angiogenesis^43^ and inflammation^44^ which may promote tumor progression^45,46^. Explaining this mechanism is outside the scope of the current study but may warrant further investigation. It is worth noting that instances of FUS-mediated BBB opening in oncologic applications, without added therapies (e.g. chemotherapy and RT), are unlikely, as diagnostic uses, such as liquid biopsies, are often performed in conjunction with therapeutic administration^47^.

The data presented in this study are preliminary and there were several study limitations. Firstly, the groups sizes (n = 4–8) are small, which made it difficult to detect small effects on tumor volume. For example, in Fig. 4b, the combined FUS + RT treatment with 8 and 15 Gy showed small decreases in the mean core tumor volumes compared with RT alone. However, due to the high variance within the groups, no statistical significance was observed. Including more data points for each set of parameters may allow better detection of small improvements. Secondly, the F98 tumor model selected for this study may not be well suited to these experiments. While F98 tumors are good models of GBM, as they are highly invasive and have low immunogenicity^48^, there are adverse characteristics of these tumors. In fact, F98 tumors are known to be resistant to radiation alone^49^, which may be linked to functionally impaired BRCA1 status^50^. In addition, F98 gliomas do not grow new blood vessels and use pre-existing blood vessels for their nutrient supply^51^. This leads to tumors with low vascular density, which poses a challenge as MB-mediated FUS therapies are targeted on the vasculature. Furthermore, the data in this study showed that, without intervention, the tumor core doubling time was approximately 2 days. This is in agreement with previous observations^28,51^. Since these tumors are more aggressive and grow more quickly than human GBM, it may be difficult to ascertain therapeutic benefit, especially in consideration of survival. Taken together, these reasons suggest that more slowly growing models of GBM, such as human patient-derived xenografts^52^, may be more suitable for future studies.

A caveat of the results presented in this study is the administration of a gadolinium MRI contrast agent following FUS sonication, and prior to RT. Previous studies have demonstrated that gadolinium-based nanoparticles can have a radiosensitizing effect, including in preclinical glioma models^53,54^ and in patients with brain metastases^55^. Such nanoparticles (≤ 5 nm diameter), which can accumulate in tumors over several hours, are preferred radiosensitizers compared gadolinium contrast agents, such as Gadavist®, which are limited by their non-targeted natures and their short tissue half lives in brain tumors (~ 20 min)^56–58^. However, Taupin, et al*.*, have demonstrated a radiosensitizing effect in in vitro F98 tumor cells, treated with the gadolinium contrast agent, Magnevist®^56^. In the present study, Gadavist® was administered, to confirm FUS-mediated BBB opening, within 1–2 h before RT, and may contribute to the effects observed on tumor growth. Future studies investigating this effect should account for this by including a study group that does not receive gadolinium prior to irradiation.

Despite limitations of group size and the tumor model, the combination of FUS and RT observed in this study did not yield a sufficient effect size to offer clinical benefit for patients with GBM. Even when FUS + RT at 4 Gy was shown to slow tumor growth compared to RT alone, the effect was not as pronounced as using RT alone at higher doses. Further work in this area should focus on optimizing the FUS and RT parameters and combining with other therapeutics to improve therapeutic outcomes. FUS parameters, such as the acoustic pressure and MB dose, may be increased. Czarnota et al. demonstrated that increased bubble doses led to an increased radiosensitization effect in a prostate cancer model, with significant increases in apoptosis with FUS and MBs alone at high MB doses^12^. The estimated dose of MBs used was 2 orders of magnitude higher than in the present study, which considered the sensitivity of the brain. It is expected that increasing MB dose would yield a larger effect, as indicated by the results in the FUS + RT compared to the FUS_BBB_ + RT groups at 15 Gy. When escalating FUS parameters, such as MB dose, caution should be taken to reduce adverse inflammatory effects in the brain^44^, such as the administration of an anti-inflammatory drug, like dexamethasone^59^. In light of the large effect on ceramide and apoptosis observed 72 h after a single treatment, multiple treatments of FUS + RT should be considered to reduce tumor burden and prolong survival. Lastly, FUS + RT may be combined with an additional therapeutic modality to improve the size of the effect. Previous research has shown that combining RT with intracerebral carboplatin or cisplatin can kill F98 tumor cells, with no residual tumor observed in surviving rats at study endpoints^39,60^. Separately, FUS-induced BBB opening has been shown to improve the delivery of systemically administered carboplatin to F98 tumors and improve survival^28^. Combining FUS, RT, and carboplatin could be an attractive avenue for future research and may allow reduced doses of carboplatin and RT to be utilized.

## Conclusion

In this study we investigated the impact of FUS + RT in the healthy rat brain and in a F98 glioma model. In the healthy brain, FUS + RT at 8 and 15 Gy was shown to have a lesioning effect. This could have implications for ablative surgery in GBM and other neurological disorders. The results of this study also demonstrate that FUS + RT may slow tumor growth, especially at low RT doses (4 Gy), where RT alone has limited benefits for tumor growth and survival. Despite the moderate impact of the combined therapy on tumor growth, there was no survival benefit. Further work is required to optimize FUS and RT parameters or to combine them with other therapeutics to improve effectiveness.

### Supplementary Information

Supplementary Information.

## Data Availability

The supporting data for in this study are available upon reasonable request by contacting Stecia-Marie Fletcher, PhD (sfletcher4@bwh.harvard.edu) or Nathan McDannold, PhD (njm@bwh.harvard.edu).
