# Supplementary material for: A study combining microbubble-mediated focused ultrasound and radiation therapy in the healthy rat brain and a F98 glioma model
Source: Sci Rep. 2024 Feb 28;14:4831. doi: 10.1038/s41598-024-55442-6 (PMC10899261; doi:10.1038/s41598-024-55442-6)
Supplement: Supplementary file 1 — Supplementary Information. [file 41598_2024_55442_MOESM1_ESM.docx]

**A Study Combining Microbubble-Mediated Focused Ultrasound and Radiation Therapy in the Healthy Rat Brain and a F98 Glioma Model**Supplemental Tables and Figures

SUPPLEMENTAL TABLE 1. Breakdown of Groups in the F98 Survival Study

| Treatment Group | Number of rats |
| --- | --- |
| Control  (no treatment) | 4 |
| FUS alone | 8 |
| RT alone  (15Gy) | 5 |
| RT alone  (8Gy) | 5 |
| RT alone  (4Gy) | 5 |
| FUS + 15Gy | 6 |
| FUS + 8Gy | 6 |
| FUS + 4Gy | 6 |
| FUS_BBB_ alone | 5 |
| FUS_BBB_ + 15Gy | 5 |


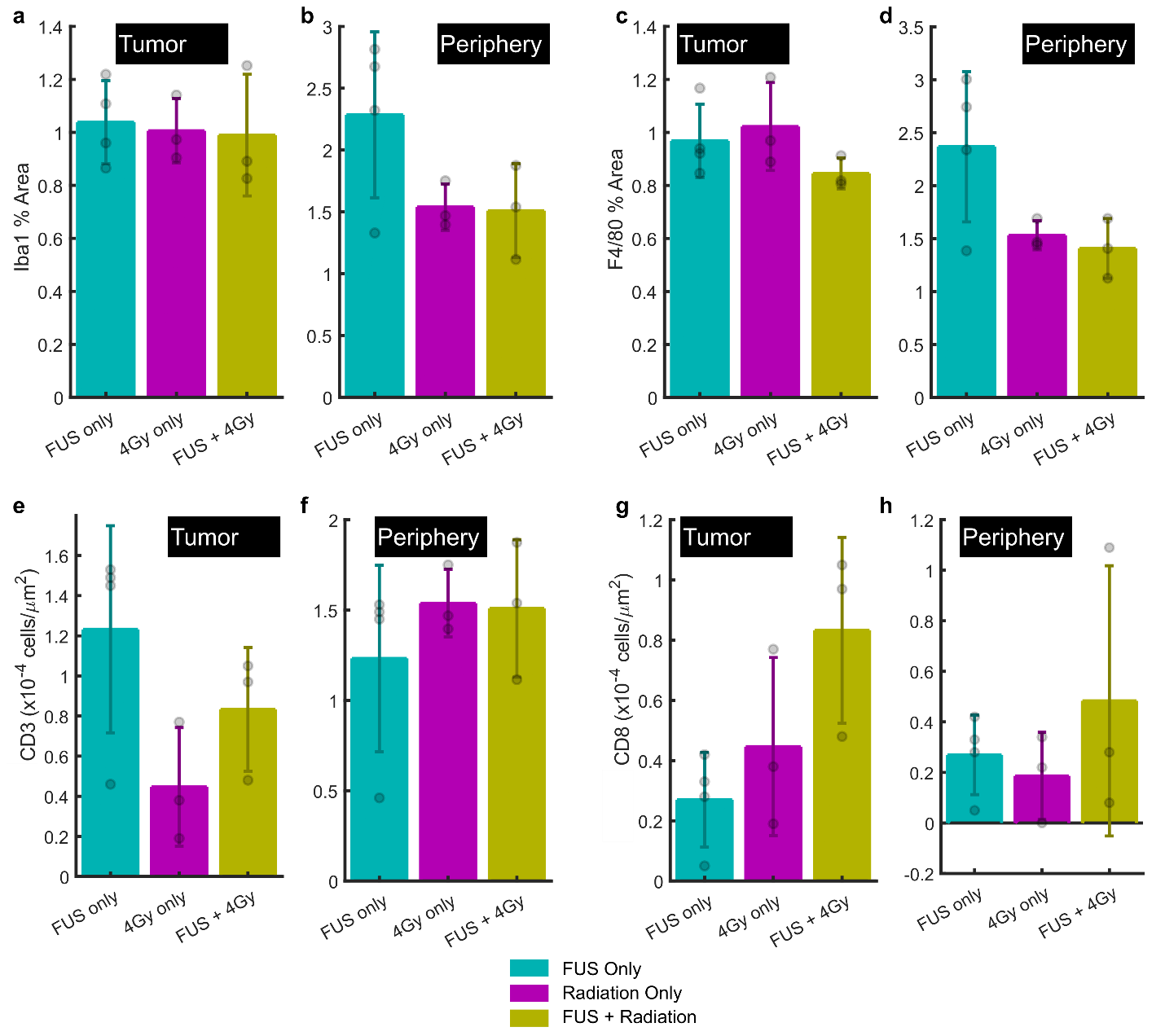


Supplemental Figure 1. Innate and adaptive immune markers at 72 hours after the treatment. a-d show no significant differences in inflammatory markers of the innate immune system (Iba1 and F4/80) which detect microglia and macrophages. e-f show no significant changes for CD3, a marker for general T-cells. g-h show no significant changes in CD8, a marker for cytotoxic T cells.
